# Knowledge, Attitude, and Practice of Colorectal Cancer Screening Among Primary Healthcare Physicians in Riyadh Second Health Cluster

**DOI:** 10.7759/cureus.32069

**Published:** 2022-11-30

**Authors:** Ahmed A Alghamdi, Abdulelah H Almutairi, Faisal S Aldosari, Abdullah M Al-Owayed, Hamza K AlOtaibi, Talal A Alghamdi, Alhanouf S Aldossary

**Affiliations:** 1 Family Medicine Consultant, Second Health Cluster, Riyadh, SAU; 2 Family Medicine Academy, King Fahad Medical City, Riyadh, SAU; 3 Family Medicine Consultant, General Directorate of Health Affairs, Riyadh, SAU; 4 Family and Community Medicine, Wadi Dawasir Hospital, Riyadh, SAU; 5 Family Medicine, Ministry of Health Holdings, Riyadh, SAU; 6 Family Medicine, Second Health Cluster, Riyadh, SAU; 7 General Practice, First Health Cluster, Ministry of Health Holdings, Riyadh, SAU; 8 Family Medicine, Second Health Cluster, King Fahad Medical City, Riyadh, SAU

**Keywords:** knowledge attitude practices studies, family physicians, knowledge score, screening guidelines, colo rectal cancer

## Abstract

Background: Colorectal cancer is the third common cancer, and the second common cause of cancer death in the world. According to the 2014 Cancer Incidence Report of the Kingdom of Saudi Arabia, colorectal cancer account for 11.5% from all cancers reported among Saudi nationals. By the year 2030, the incidence of colorectal cancer could increase fourfold among both genders.

Aim: The study aimed to conduct to understand the knowledge, attitude, and practice of primary healthcare physicians regarding colorectal cancer screening in Riyadh Second Health Cluster in the city of Riyadh, Saudi Arabia.

Materials and methods: A cross-sectional study design, probability proportional to size sampling at the cluster zones level and convenient sampling for the physicians, were used among physicians working in the primary healthcare centers in the city of Riyadh between October 2022 and November 2022.

Result: Of the 213 respondents, the mean age of the physician was 35, the majority were males (59%), Saudi Arabian nationality (60%), Resident Physicians (54%) and ≤ five years’ work experience (45%). Sixty-four percent of the study participants believe that the majority of asymptomatic average-risk patients have to start the screening at the age of 45 years old. Ninety-seven percent believe that colorectal cancer screening for asymptomatic average-risk patients aged 45 years and older is effective. Ninety-two percent of the physicians perform colorectal cancer screening for asymptomatic average-risk patients aged 45 years. The mean knowledge score is 4.65 (SD=2.33) with a range of 0 to 10. The mean attitude score is 4.19 (SD=1.28) with a range of 0 to 6.

Conclusion: The study found that the physicians had higher attitude and practice towards colorectal cancer screening and adequate knowledge towards colorectal cancer screening. The knowledge and attitude scores are associated with practicing colorectal cancer screening.

## Introduction

Colorectal cancer (CRC) is cancer that affects the colon or rectum [1]. In the colon or rectum, an abnormal growth, polyps, can occasionally develop before cancer [1,2]. Colorectal cancer has the highest rate of incidence in developed countries [3]. As the general population moves more towards a sedentary lifestyle, overweight and obesity, low diet in fruits and vegetables, smoking, and heavy alcohol use, it increases the prevalence of colorectal cancer rapidly [2]. Around 90% of colorectal cancer cases occur in the 50 years and older population. The other non-modifiable risk factors include inflammatory bowel disease, family history and genetic syndrome like familial adenomatous polyposis external or hereditary non-polyposis colorectal cancer [1,2].

Colorectal cancer screening tests like guaiac-based fecal occult blood test (gFOBT), fecal immunochemical test (FIT), flexible sigmoidoscopy, colonoscopy, and CT colonography can find polyps and early stages of colorectal cancer, hence, appropriate intervention can be initiated at an early stage [1].

Diagnosis at early stage and treatment of cancer can improve outcomes and reduce mortality [4]. Thus, screening for cancer is essential to detect asymptomatic persons with particular cancers and to refer them for diagnosis and early treatment [4]. The median age at diagnosis was 60 years in males and 57 years in females. The recommended screening for colorectal cancer according to national guidelines of the Ministry of Health and guidelines of the American Cancer Society and the US Preventative Services Task Force (USPSTF) starts at age 45 years [5-7]. Recommended screening strategies include annual screening with high-sensitivity FOBT; screening every five years with flexible sigmoidoscopy, or screening with colonoscopy every 10 years [6]. The Saudi expert panel suggests not using colorectal cancer screening for asymptomatic persons at average risk aged 70 years or older [7].

Cancer is the second leading cause of mortality after cardiovascular disease [5]. In the year 2015, its incidence was 17.5 million and mortality was 8.7 million cases worldwide [8]. Colorectal cancer is the third most common cancer after lung and breast cancer worldwide, and the second most common cause of cancer death in the world [4]. Among men, it is the second leading cause of cancer death after lung cancer, and among women the third leading cause of cancer death after breast cancer and lung cancer [5,9]. From 2005 to 2015, colorectal cancer had the fourth highest absolute years of life lost among all types of cancer [5]. In 2015, the incidence of colorectal cancer was 1.7 million, and it caused 832,000 deaths worldwide [8]. It is estimated that in the year 2030, there will be 2.2 million incidences and 1.1 million colorectal cancer-related mortality [10].

Colorectal cancer is the most common cancer among Saudi men and the third most common among Saudi women with a constant rise in the incidence for the past few years [11]. According to the 2014 Cancer Incidence Report of the Kingdom of Saudi Arabia (KSA), colorectal cancer accounted for 11.5% of all cancers reported among Saudi nationals [12]. The Riyadh region has the highest incidence rate among females and second highest among males [12]. The incidence of colorectal cancer in KSA has been on a constant rise over the past few years due to possible Westernization of dietary habits and lack of proper screening [13]. The age at the time of diagnosis is lower compared with developed countries [13].

“Past, Present, and Future of Colorectal Cancer in the Kingdom of Saudi Arabia” study was conducted by Ibrahim et al. in the year 2008. The authors compared the colorectal cancer statistics (from 1994 to 2003) from the National Cancer Registry of KSA with the statistics of the United States of America (USA). They demonstrated that the age-standardized rates of colorectal cancer statics doubled from 1994 to 2003, in contrast to the USA where the rates were progressively declining [14]. The authors mentioned that the rate decline in the USA was associated with screening and early removal of the polyps [15]. On the other hand, in KSA, there was a progressive increase in exposure to risk factors and lack of a nationwide screening program. The authors also estimated that by the year 2030, the incidence of colorectal cancer could increase fourfold among both genders [14].

In 2015, Almadi et al. conducted a cross-sectional survey in Riyadh, KSA, to understand the public knowledge, attitudes, and behavior regarding willingness to undergo colorectal cancer screening using the Health Belief Model (HBM) [16]. Five hundred participants were involved in the survey, among them 35.6% of the study population mentioned that colorectal cancer is common [16]. The mean knowledge score was 10.50 (SD=4.4) with the range of lowest score by 2 to the highest score of 23 [16]. The most commonly recognized tool for screening technique was colonoscopy (50.56%), followed by CT colonography (32.7%), stool-based screening (24.7%), whereas the least appreciated method was flexible sigmoidoscopy (14.7%) [16].

In 2019, the gap between knowledge and colorectal cancer screening using the HBM in 2019: A national survey was conducted by Almadi and Alghamdi [17]. It was a huge nationewide survey throughout the KSA in all 13 jurisdictions. 5720 individuals’ responded to the survey via an electronic platform [17]. 15.24% of the study participants had already undergone colorectal cancer screening using various screening techniques, mostly colonoscopy (72.73%). The mean knowledge score was 11.05 (SD=4.4) with a range of 1 to 23. Survey participants had a positive attitude toward colorectal cancer screening as well as to colonoscopy screening technique, and 73% expressed willingness to undergo screening in the future. Even though the majority of participants responded with willingness to undergo screening, no significant correlation was found between knowledge and willingness to undergo screening were predicted. Moreover, the majority of the participants mentioned their interest in screening in the survey, on the other hand, the majority of the participants refused the invitation to undergo screening [17].

Federici et al. conducted a survey on colorectal cancer screening knowledge, attitudes, and practices of general practice physicians in Lazio, Italy in the year 2006 among 699 General Practitioners (GPs). Ninety-four percent mentioned that colorectal cancer could be prevented. GPs who use guidelines in their practice had statistically significantly higher knowledge in oncological screening. Only 25% of the study participants recommended colorectal cancer screening to their patients. The authors mentioned that the “knowledge about screening and use of guidelines as sources of scientific information are important factors to improve attitudes about screening” [18].

In Saskatchewan, 773 family physicians were included in a survey conducted by Deobald et al. to evaluate the current colorectal cancer screening practices and identify barriers to screening to improve current practice. The authors mentioned that the colorectal cancer screening recommended by a family physician was occurring at a low rate [19]. The waiting time for colonoscopy and access are the main barriers to colorectal cancer screening [19]. The authors mentioned that recently introduced FIT test in the province should improve the overall screening but will require further increases in resources and availability of colonoscopy for follow-up of positive results [19].

In Riyadh, KSA, in the year 2014, Demyati conducted a study to “explore the current knowledge, attitude, and practice of family physicians working in family medicine clinics in National Guard Health Affairs (NGHA), Riyadh, toward colorectal cancer screening and to identify the barriers of the screening” [20]. One hundred seventy family physicians were included, the mean knowledge score was 5.02 (SD=2.73) with a range from 0 to 11. Board-certified physicians had higher knowledge scores than other physicians [20]. 94.6% of the physicians considered that the screening for asymptomatic average-risk patients is effective and 81.5% of the physicians prefer structured screening programs over opportunistic screening programs [20]. The mean attitude score was found to be 6.95 (SD=1) and the range was between 4 and 8. More than half (56.2%) of the physicians are not practicing colorectal cancer screening for asymptomatic average-risk patients [20]. The author concluded that the large proportion of family physicians in the study do not recommend colorectal cancer screening, even though the knowledge level and the positive attitude [20].

A study was conducted in Washington State to understand colorectal cancer screening practices of primary care physicians, by Hannon et al [21]. Seventy-six percent of primary healthcare physicians recommended one or more colorectal cancer screening tests in agreement with American Cancer Society guidelines [21]. And the physicians perceived that colorectal cancer screening is essential. The author mentioned that “educating physicians about the power of their recommendations to affect patient behavior may encourage physicians to recommend colorectal cancer screening to more of their patients strongly” [21]. The author also recommended three interventions to improve colorectal screening [21]. The recommendations are educating primary care physicians about screening guidelines, encouraging physicians to discuss colorectal cancer screening with their patients and improving physicians’ capacity to track and follow colorectal cancer screening to completion [21].

Studies show that the main reasons for colorectal cancer deaths are mainly a lack of awareness in the population, financial barriers, and lack of knowledge of colorectal cancer screening guidelines among healthcare physicians and nurses [16,17,22]. Also, a majority of the study authors suggest that early detection of colorectal cancer and educational campaigns plays a huge role in preventing colorectal cancer mortality rate [17,22]. Primary healthcare physicians have an important role in screening practice due to frequent contact with a large population of patients. The involvement of primary healthcare physicians in screening implementation has been recommended by several guidelines [20].

Several studies assessed the knowledge of colorectal cancer and its screening among the general public in KSA [16,17]. And a study conducted by Demyati explored the current knowledge, attitude, and practice of family physicians working in family medicine clinics in National Guard Health Affairs (NGHA), Riyadh [20]. KSA lacks the study knowledge, attitude, and practice of colorectal cancer screening among primary healthcare physicians.

Based on the gap in the literature, the study aimed to understand the knowledge, attitude, and practice of primary healthcare physicians regarding colorectal cancer screening in Riyadh, Saudi Arabia.

The specific research objectives are as follows:

1. To assess the current knowledge and attitude of physicians working in primary healthcare centers of Riyadh Second Health Cluster in the city of Riyadh toward screening for colorectal cancer.

2. To determine the practice and adherence to national or international screening guidelines among physicians working in primary healthcare centers of Riyadh Second Health Cluster in the city of Riyadh toward screening for colorectal cancer.

3. To identify the current barriers to implementing colorectal cancer screening recommendations in primary healthcare centers of Riyadh Second Health Cluster in the city of Riyadh to improve current practice.

## Materials and methods

Study design

A cross-sectional descriptive study design was used to assess the knowledge, attitude, and practice among primary healthcare physicians working in Riyadh Second Health Cluster in the city of Riyadh, Saudi Arabia between October 2022 and November 2022 regarding colorectal cancer screening. 

Study setting and population

The study participants are primary healthcare physicians working under the primary healthcare centers of Riyadh Second Health Cluster in the city of Riyadh in the five zones (zone one, two, three, four, and five) of Riyadh city.

Inclusion criteria

Physicians working in Primary Healthcare Centers (PHC) of Riyadh Second Health Cluster in the city of Riyadh who are practicing as residents, registrars, or consultants. 

Exclusion criteria

Physicians not available on the date of data collection, physicians on vacation, or physicians who refused consent.

Sample size estimation

A total number of 356 primary healthcare physicians were registered with Riyadh Second Health Cluster in the city of Riyadh at the time we conducted our survey. The sample size was calculated assuming the prevalence of 44% (according to a study conducted by Demyati that reported that 56.2% of the physicians are not practicing colorectal cancer screening for asymptomatic average-risk patients), a precision of 7%, an alpha level of 5%, assuming non-responding physicians 10%, the calculated minimum sample size is 170 physicians [20]. 

Sampling technique

The sampling technique is probability proportional to size sampling at the cluster zones (five zones in the city of Riyadh) level. A list of total number of physicians in each zone was obtained from the Riyadh Second Health Cluster and PPS was applied to get the number of physicians to be selected from each sector. This was 43 for the Zone One, 36 for the Zone Two, 30 for the Zone Three, 27 for the Zone Four, and 34 for the Zone Five. Then, four to six of the largest PHCs were selected in each zone. Then, depending on the sample required, the number of physicians who met the inclusion criteria from each PHC was selected conveniently.

Data collection tools

The questionnaire contains questions regarding the demographic characteristics of the physician and cancer screening knowledge, attitude, current practice, and barriers to practicing screening of colorectal cancer of the physician. The items of the questionnaire were adapted from a validated questionnaire of a survey conducted by Demyati in 2014 and entitled “Knowledge, Attitude, Practice, and Perceived Barriers of Colorectal Cancer Screening among Family Physicians in National Guard Health Affairs, Riyadh” [20]. These items were adapted with permission from the author and were customized by adding and eliminating questions to be in line with the characteristics of PHCs of Riyadh Second Health Cluster. A pilot study was conducted on 10 physicians to check understanding of the questionnaire. The questionnaire contains the following domains:

Domain 1: demographic characteristics:

Age, Gender, Nationality (Saudi, Non-Saudi), Medical degree, Zone and Years in practice.

Domain 2: Knowledge and attitude of colorectal cancer screening:

Starting age for screening, Stopping age for screening, Appropriate screening interval, Appropriate screening method, Efficacy of screening, and Efficacy of screening method. 

Domain 3: Practice of colorectal cancer screening:

Routinely recommending screening, Screening method most often discussed and Referring patient for screening. 

Domain 4: Barriers to colorectal cancer screening:

Uncertainty about efficacy, Insufficient consultation time, Patient believes screening not effective, Patient unaware of CRC screening and Lack of policy or procedure in the workplace.

Data management and statistical analysis plan

Data Management

All data from the questionnaire were entered into an Excel spreadsheet (Microsoft, Redmond, WA, USA) and to check for the accuracy of the entered data, randomly 10% of the questionnaire was selected and rechecked. For statistical analysis we used SPSS v.20 (IBM Corp., Armonk, NY, USA). 

Variables

The primary outcome was the practice of colorectal cancer screening among PHC physicians. Secondary outcome variables included the knowledge and attitude scores of the physicians. The variables like age, gender, nationality, medical degree, zone, and years in practice are considered control variables according to the literature.

Statistical Analysis

The descriptive analysis was performed and presented demographic data of the physicians. Continuous variables were presented by means and standard deviations, and knowledge and attitude scores are compared with control variables using independent t-tests and ANOVA. Categorical and ordinal variables were presented by percentages, and the primary outcome variable practice was compared with control variables using Chi-square (χ2) tests. 

The knowledge score was computed by giving one point to the correct answers and zero points to the wrong answers and for the option “not sure.” The items included for knowledge score calculation are as follows, 45 years of age as starting age for asymptomatic average-risk patients screening, aware about stopping age for screening, aware about FOBT types Guaiac FOBT and FIT, aware about FOBT office card and FOBT home kit, ordering two samples for each FOBT, FBOT screening annually, flexible sigmoidoscopy screening every five years, and colonoscopy screening every 10 years. There were 10 items, with a minimum score of zero and a maximum score of 10. The items included in the knowledge score are based on the prior study [20].

The attitude score was computed by giving one point for the correct answer and zero points for the wrong answers and the option “not sure.” The items included for attitude score calculation are as follows: CRC screening is effective for the asymptomatic average-risk patient, FOBT is effective, flexible sigmoidoscopy is effective, colonoscopy is effective, double-contrast barium enema is effective, and CT-colonography is effective. There were six items, with a minimum score of 0 and a maximum score of 6. The items included in the attitude score are based on the prior study [20]. 

Ethical considerations

This study was conducted with the ethical standards mentioned by the Ministry of Health (MOH) and the local Institutional Review Board (IRB) Ethical approval for this study was obtained from King Fahad Medical City IRB, Saudi Arabia (approval 22-498E). Consent was obtained from all participants before participating in the study. Participants were informed of their right to refuse participation or withdrawal from the study at any time without any penalties or consequences. The participants were made aware of the anonymity of their participation and informed that under no circumstances will any of their personal identifying information be collected or revealed or published. No incentive was given to the participants for participation. All the data collection forms were kept under strict confidentiality, accessible only to the researcher. The study did not anticipate any harm to the participants as a result of participating in the study.

## Results

Demographic statistics

Overall, 213 participants were included in the study, among them, 37% from Zone One, 23% from Zone Two, 13% from Zone Three, 11% from Zone Four, and 16% from Zone Five. The mean age of the study participants was 35.34 (SD=8.56) with the minimum and maximum age range of the physicians being 25 and 62, respectively. Among them, 59% were male, and 41% were female. The majority of the physicians were considered to be Saudi Arabian nationality with a percentage of 60%. Fifty-four percent of the physicians were reported as residents, 38% as registrars and the rest (8%) as consultants. Experience of the physicians with less than or equal to five years, six to 10 years, 11 to 15 years and more than 15 years of experience are 45%, 27%, 11%, and 16% respectively (Table 1).

**Table 1:** Demographic data of the study participants

Variable		Mean	Range
Age		35.34	25 - 62
Variables	Categories	N	%
Gender	Female	87	41%
	Male	126	59%
Nationality	Non-Saudi	85	40%
	Saudi	128	60%
Medical Degree	Consultant	17	8%
	Registrar	81	38%
	Resident	115	54%
Zone	Zone One	80	38%
	Zone Two	48	23%
	Zone Three	27	13%
	Zone Four	23	11%
	Zone Five	35	16%
Years in practice	>15	35	16%
	≤ 5	96	45%
	11-15	24	11%
	6-10	58	27%

Knowledge of the physicians regarding colorectal cancer screening

Sixty-four percent of the study participants believe that the majority of asymptomatic average-risk patients have to start the screening at the age of 45 years old. The majority of the physicians (60%) also believe that screening for a healthy population is no longer recommended after a particular age, the mean age is 72.97 with the range of 50 to 90 years old.

As per the physician's knowledge, the frequency of screening with FOBT is one year (45%), two years (19%), three years (19%) and not sure (17%). Forty-seven percent of the physicians are aware of the Guaiac FOBT type, and 46% are aware of the fecal immunochemical testing FOBT type. In the ways of conducting FOBT tests, 35% of the physicians were aware the FOBT card can be done in the office during the digital rectal examination, and 48% of the physician agree that the FOBT kits can be given to the patients to do the test at home. For colorectal cancer screening using FOBT, 8% of the physician said that one sample should be ordered, 42% for two samples, 23% for three samples and 28% are not sure about several samples to be collected. 

For the assessment of knowledge about the frequency of screening with sigmoidoscopy 2% of the physician answered it should be done every one year, 28% are for every three years, the majority (51%) are for every five years, 7% for every 10 years and 125 are not sure. For colonoscopy, the majority (46%) answered it should be done every 10 years (Table 2).

**Table 2:** Knowledge of the colorectal cancer screening of the study participants FOBT: fecal occult blood test

Variables	Categories	N	%
Start of screening at the age of asymptomatic average-risk patients	45 years	136	64%
	50 years	52	24%
	55 years	17	8%
	60 years	0	0%
	Not sure	8	4%
Is there an age at which no longer recommend screening for healthy patients?	No	85	40%
	Yes	128	60%
	If yes	Average age	Range
		72.97	50 - 90
The frequency of screening with fecal occult blood test is	Not sure	36	17%
	One year	95	45%
	Three years	41	19%
	Two years	41	19%
Aware of the following types of FOBT			
Guaiac FOBT	No	112	53%
	Yes	101	47%
Fecal Immunochemical Testing	No	114	54%
	Yes	99	46%
Aware of the following means of conducting FOBT			
FOBT card in the office during a digital rectal exam	No	138	65%
	Yes	75	35%
Give patients FOBT kits to complete at home	No	110	52%
	Yes	103	48%
For Colorectal Cancer Screening using FOBT, how many samples do you suppose to order	One	16	8%
	Two	90	42%
	Three	48	23%
	Not sure	59	28%
The frequency of screening with Sigmoidoscopy is every	1 year	5	2%
	3 years	60	28%
	5 years	108	51%
	10 years	15	7%
	Not sure	25	12%
The frequency of screening with Colonoscopy is every	1 year	8	4%
	3 years	20	9%
	5 years	61	29%
	10 years	97	46%
	Not sure	27	13%

Attitude of the physicians regarding colorectal cancer screening

The vast majority of the physicians, around 97%, believe that colorectal cancer screening for asymptomatic average-risk patients aged 45 years and older is effective. For the following screening procedures fecal occult blood testing, flexible sigmoidoscopy, colonoscopy, double-contrast barium enema, and CT-colonography, 80%, 82%, 91%, 25% and 35% of the physicians respectively believe that it reduces colorectal cancer mortality in average-risk patients aged 45 years and older (Table 3).

**Table 3:** Attitude on the colorectal cancer screening of the study participants

Variable	Category	N	%
Colorectal Cancer Screening for asymptomatic average-risk patients aged 45 years and older is effective	No	7	3%
	Yes	206	97%
The following screening procedures are in reducing colorectal cancer mortality in average-risk patients aged 45 years and older	Don’t know	Effective	Not-effective
Fecal Occult Blood Testing	20 (9%)	170 (80%)	23 (11%)
Flexible sigmoidoscopy	27 (13%)	175 (82%)	11 (5%)
Colonoscopy	18 (08%)	192 (91%)	3 (01%)
Double-Contrast Barium enema	85 (40%)	54 (25%)	74 (35%)
CT-Colonography	86 (40%)	95 (45%)	32 (15%)

Practice of the physicians in colorectal cancer screening

Almost 92% of the physicians perform colorectal cancer screening for asymptomatic average-risk patients aged 45 years and older. Among them, 90% of physicians prefer a structured screening program to conduct colorectal cancer screening, and overall, 77% of physicians prefer a structured screening program. During a typical month, the majority of the physicians (55%) order or perform FOBT screening tests one to five times. In contrast to FOBT, during a typical month, 10% of the physicians refer asymptomatic, average-risk patients for screening sigmoidoscopy, or colonoscopy one to five times. Ninety percent of the physicians do not order or perform sigmoidoscopy or colonoscopy for asymptomatic or average-risk patients (Table 4).

**Table 4:** Practice of the colorectal cancer screening of the study participants

Variables	Categories	N	%
Perform Colorectal Cancer screening for asymptomatic average-risk patients aged 45 years and older	No	18	8%
	Yes	195	92%
Following ways of conducting colorectal cancer screening do you prefer	Opportunistic screening	48	23%
	Structured screening program	165	77%
During a typical month, how many times do you order or perform this screening test (FOBT)	0 times	78	37%
	1-5 times	118	55%
	6-10 times	14	7%
	11-20 times	2	1%
	>20	1	0%
During a typical month, how many times do you refer asymptomatic, average-risk patients for screening Sigmoidoscopy or Colonoscopy	0 times	192	90%
	1-5 times	21	10%
	6-10 times	0	0%
	11-20 times	0	0%
	>20	0	0%

Fecal occult blood test, sigmoidoscopy, colonoscopy and others from the above-mentioned screening test or with the combination of the screening tests, majority of the physicians (45%) mentioned that they usually discuss FOBT screening tests with their patients, only 4% discuss sigmoidoscopy and 17% of the physician discuss all three screening tests to their patients (Table 5).

**Table 5:** Which of the following screening tests do you usually discuss with your patients?

Variables	N	%
Fecal Occult Blood Test	96	45%
Sigmoidoscopy	8	4%
Colonoscopy	24	11%
Other	3	1%
Sigmoidoscopy;Colonoscopy	4	2%
Fecal Occult Blood Test; Colonoscopy	29	14%
Fecal Occult Blood Test; Sigmoidoscopy	10	5%
Fecal Occult Blood Test; Other	1	0%
Fecal Occult Blood Test; Sigmoidoscopy; Colonoscopy	37	17%
Fecal Occult Blood Test; Sigmoidoscopy; Colonoscopy; Other	1	0%

Barriers to recommending colorectal cancer screening

Table 6 summarizes the barriers to recommending colorectal cancer screening. The barriers like patients being unaware of colorectal cancer screening (48%), patients do not follow through to complete colorectal cancer screening tests (60%), there being no policy and procedure in the workplace for screening (45%) and there being no reminder system in the workplace (45%) are reported that it usually occurs among the majority of the physicians. The other majority mention that the following barriers sometimes occur, not having enough time to discuss screening with patients (45%), patients do not want to discuss colorectal cancer screening (45%), patients having difficulty understanding the information present about colorectal cancer screening (51%), and patients not perceiving colorectal cancer as a serious health threat (48%).

**Table 6:** Barriers to colorectal cancer screening for asymptomatic, average-risk patients in your practice

Barriers	Never	Rarely	Sometimes	Usually
Not having enough time to discuss screening with my patients	38 (18%)	46 (22%)	95 (45%)	34 (16%)
My patients do not want to discuss colorectal cancer screening	38 (18%)	58 (27%)	95 (45%)	22 (10%)
My patients have difficulty understanding the information I present about colorectal cancer screening	29 (14%)	59 (28%)	109 (51%)	16 (8%)
My patients are unaware of colorectal cancer screening	12 (6%)	23 (11%)	76 (36%)	102 (48%)
My patients do not perceive colorectal cancer as a serious health threat	23 (11%)	45 (21%)	102 (48%)	43 (20%)
My patients do not follow through to complete colorectal cancer screening tests	5 (2%)	10 (5%)	70 (33%)	128 (60%)
There is no policy and procedure in my work-place for screening	13 (06%)	29 (14%)	76 (36%)	95 (45%)
There is no reminder system in my work-place	6 (03%)	24 (11%)	88 (41%)	95 (45%)

Knowledge score and its comparison with control variables

The mean knowledge score is 4.65 (SD=2.33), with a range from 0 to 10. The knowledge score is compared with all of the control variables. None of the control variables age (p-value=0.311), gender (p-value=0.101), nationality (p-value=0.593), zone (p-value=0.511), and years in practice (p-value=0.311) showed statistically significant mean difference in the knowledge score except the variable medical degree, resident physicians had significantly lesser mean 3.88 (SD=2.17) in comparison with consultant physicians mean 6.35 (SD=2.23) and registrar physicians mean 5.38 (SD=2.15) with the P-value <0.001 (Table 7).

**Table 7:** Analysis of variance (ANOVA): Knowledge score compared with the demographic characteristics of the physician

Variables	Categories	Mean (SD)	95% CI for Mean		F	P-value
			Lower	Upper		
Age	≤ 40	4.56 (2.35)	4.20	4.92	01.03	.311
	>40	4.95 (2.23)	4.29	5.62		
Gender	Female	4.33 (2.27)	3.84	4.82	02.70	.101
	Male	4.86 (2.35)	4.45	5.28		
Nationality	Non-Saudi	4.75 (2.07)	4.31	5.20	00.29	.593
	Saudi	4.58 (2.49)	4.14	5.01		
Medical Degree	Consultant	6.35 (2.23)	5.20	7.50	17.15	.000
	Registrar	5.38 (2.15)	4.91	5.86		
	Resident	3.88 (2.17)	3.48	4.28		
Zone	Zone One	4.90 (2.40)	4.36	5.43	00.84	.511
	Zone Two	4.47 (2.21)	3.86	5.09		
	Zone Three	4.07 (2.81)	2.96	5.19		
	Zone Four	4.96 (1.82)	4.17	5.74		
	Zone Five	4.54 (2.14)	3.75	5.34		
Years in practice	≤ 5	4.29 (2.44)	3.74	4.69	02.15	.095
	6-10	5.12 (2.34)	4.50	5.77		
	11-15	5.00 (2.36)	4.00	5.10		
	>15	4.80 (2.10)	4.08	5.52		

Attitude score and its comparison with control variables

The mean attitude score is 4.19 (SD=1.28), with a range from 0 to 6. The participants aged >40 years old had significantly higher mean attitude score 4.63 (SD=1.25) compared to those aged ≤40 years (p-value=0.008). In the variable years in practice, ≤ five years had significantly lesser mean attitude score 3.99 (SD=1.15) compared with the >15 years in practice mean score 4.71 (SD=1.18). The other control variable gender (p-value=0.095), nationality (p-value=0.510), medical degree (p-value=0.117), and zone (p-value=0.431), did not show statistically significant mean difference in the attitude score (Table 8).

**Table 8:** Analysis of variance (ANOVA): Attitude score compared with the demographic characteristics of the physician

Variables	Categories	Mean (SD)	95% CI for Mean		F	P-value
			Lower	Upper		
Age	≤ 40	4.06 (1.26)	3.87	4.26	07.24	.008
	>40	4.63 (1.25)	4.26	5.00		
Gender	Female	4.01 (1.35)	3.72	4.29	02.82	.095
	Male	4.30 (1.21)	4.09	4.52		
Nationality	Non-Saudi	4.26 (1.43)	3.95	4.57	00.44	.510
	Saudi	4.14 (1.17)	3.94	4.35		
Medical Degree	Consultant	4.53 (1.07)	3.98	5.08	02.16	.117
	Registrar	4.35 (1.42)	4.03	4.66		
	Resident	4.03 (1.18)	3.81	4.24		
Zone	Zone One	4.20 (1.28)	3.92	4.48	00.96	0.431
	Zone Two	4.40 (1.35)	4.00	4.79		
	Zone Three	4.07 (1.36)	3.54	4.61		
	Zone Four	3.78 (1.35)	3.20	4.37		
	Zone Five	4.23 (1.06)	3.86	4.59		
Years in practice	≤ 5	3.99 (1.15)	3.76	4.22	2.83	.040
	6-10	4.21 (1.41)	3.84	4.58		
	11-15	4.17 (1.43)	3.56	4.77		
	>15	4.71 (1.18)	4.31	5.12		

Practice in comparison with control variables

Almost 92% of the physicians perform colorectal cancer screening for asymptomatic average-risk patients aged 45 years and older. In comparing practice with other control variables, none of the control variables showed a statistically significant difference. The variables are age (p-value=0.595), gender (p-value=0.745), nationality (p-value=0.681), medical degree (p-value=0.529), zone (p-value=0.643) and years in practice (p-value=0.875). Table 9 summarizes the Chi-Square analysis for practice and its comparison with control variables.

**Table 9:** Chi-Square: Practice and its comparison with control variables

Variables	Categories	Practicing (% within practice)	χ2	P-value
Age	≤ 40	77.9%	0.282	.595
	>40	22.1%		
Gender	Female	40.5%	0.105	.745
	Male	59.5%		
Nationality	Non-Saudi	39.5%	0.169	.681
	Saudi	60.5%		
Medical Degree	Consultant	8.2%	1.273	.529
	Registrar	39.0%		
	Resident	52.8%		
Zone	Zone One	39.0%	2.507	.643
	Zone Two	22.6%		
	Zone Three	12.3%		
	Zone Four	10.3%		
	Zone Five	15.9%		
Years in practice	≤ 5	45.1%	0.692	.875
	6-10	27.7%		
	11-15	10.8%		
	>15	16.4%		

Univariate logistic regression analysis

To predict the association of independent variable (knowledge score and attitude score) with the dependent variable (practicing colorectal cancer screening), we ran a univariate logistic regression analysis. In the analysis, the knowledge score is associated with practicing the colorectal cancer screening (odds=1.310 and the p-value=0.019), which possibly explains that the physicians with higher knowledge scores are more likely to practice colorectal cancer screening. The attitude score is associated with practicing colorectal cancer screening (odds=1.404 and the p-value=0.049), which possibly explain that the physicians with higher attitude scores are more likely to practice colorectal cancer screening (Table 10).

**Table 10:** Univariate regression analysis to predict independent and dependent variable (practicing colorectal cancer screening) association

Independent Variable	Unadjusted odds ratios	95% CI for odds		P-value
		Lower	Upper	
Knowledge score	1.310	1.045	1.642	0.019
Attitude score	1.404	1.002	1.969	0.049

## Discussion

The study assessed the knowledge, attitude, and practice of primary healthcare physicians regarding colorectal cancer screening in Riyadh Second Health Cluster in the city of Riyadh, Saudi Arabia. Using a cross-sectional survey. We surveyed 213 physicians working in PHC in five zones of the city of Riyadh. 

The mean age of the physician was 35, the majority were males (59%), Saudi Arabian nationality (60%), resident physicians (54%) and ≤ five years’ work experience (45%). There were some differences in the characteristics of the study participants in comparison with other similar studies. In the survey conducted among family physicians in NGHA the majority were females (52%) and the mean age was 38 years old [20]. The study conducted in Saskatchewan among family physicians had 50% of the physicians practicing for around six to 10 years [19].

In the study conducted in Saskatchewan [19], 79.9% of respondents recommending screening beginning at age 50 and 78.9% for the previous study conducted in KSA [20], Riyadh comparatively in our study only 64% of the study participants believe that the majority of asymptomatic average-risk patients, have to start the screening at the age of 45 years old. Around 47% were aware of FOBT types, Guaiac FOBT and 46% FIT, 35% were aware of FOBT office card and 48% of FOBT home kit, 42% were aware of ordering two samples for each FOBT, 45% of the responded for FBOT screening annually, 51% were aware of flexible sigmoidoscopy screening every five years, and 46% aware of colonoscopy screening every 10 years. The study is more or less consistent with the study finding conducted in NGHA [20] 

The majority of the participants in our study had a positive attitude towards colorectal cancer screening, which is consistent with the other studies, except for the screening method of double-contrast barium enema and CT-colonography; only 25% and 35% of the physicians respectively believe that they reduce colorectal cancer mortality in average-risk patients aged 50 years and older. 

Ninety-two percent of the physicians perform colorectal cancer screening for asymptomatic average-risk patients aged 50 years and older contrasting with the previous study in Riyadh where 50% perform colorectal cancer screening [20]. None of the control variables had a significant association with the practice whereas the previous study conducted in Riyadh male participants and physicians aged >40 significantly higher colorectal cancer screening practice. In our study, 45% mentioned that they usually discuss FOBT screening tests with their patients in contrast with the study conducted in Saskatchewan where 71.8% recommend FOBT and 80.8% for the previous study conducted in Riyadh [19,20]. The most preferred screening method in our study is FOBT whereas in the study conducted in Saskatchewan colonoscopy was the most preferred method [19]. 

The barriers like patient unawareness of colorectal cancer screening (48%), patients do not follow through to complete colorectal cancer screening tests (60%), no policy and procedure in the workplace for screening (45%) and no reminder system in the workplace (45%) are reported that it usually occurs among the majority of the physicians.

The mean knowledge score is 4.65 (SD=2.33) with a range of 0 to 10. None of the control variables showed a statistically significant mean difference in the knowledge score except the variable medical degree; Consultant mean 6.35 (SD=2.23) and Registrar mean 5.38 (SD=2.15) had significantly higher knowledge scores. The mean attitude score is 4.19 (SD=1.28), the participants aged >40 years old and >15 years in practice had significantly higher mean attitude score 4.63 (SD=1.25) and 4.71 (SD=1.18) respectively.

Strengths and limitations

The study utilized a questionnaire from a previously conducted study, which increases the validity of the questionnaire. The study included physicians from all zones of the Riyadh Second Health Cluster in the city of Riyadh, increasing the generalizability of the study findings to the Riyadh city. However, the study cannot be generalized to the whole Kingdom because it is conducted only in Riyadh city. 

## Conclusions

The majority of the study participants believe that the mainstream of asymptomatic average-risk patients have to start screening at the age of 45 years old. Majority of the physicians believe that colorectal cancer screening for asymptomatic average-risk patients aged 45 years and older is effective, and the physicians perform colorectal cancer screening for asymptomatic average-risk patients aged 45 years. The study found that the physicians had higher attitudes and practice towards colorectal cancer screening and satisfactory knowledge about the screening guidelines. The knowledge and attitude scores are associated with practicing colorectal cancer screening; in other words, the physicians with higher knowledge and attitude scores are more likely to practice colorectal cancer screening.

Based on the study findings, physicians need frequent seminars and continuing medical education programs on colorectal cancer screening. We recommend implementing policies and a reminder system to practice colorectal cancer screening. Furthermore, the patient's knowledge and willingness to be screened for colorectal cancer are important factors in practicing colorectal cancer screening. Therefore, an educational program should be initiated for the general population.

## Appendices

APPENDIX 1: SURVEY QUESTIONNAIRE

Knowledge, Attitude, and Practice of Colorectal Cancer Screening among Primary Healthcare Physicians in Riyadh Second Health Cluster

Please answer each of the following questions by ticking the appropriate choice:

Part I: Demographic Data

1.     Age: _______ years

2.     Gender

      Male 

      Female

3.     Nationality

      Saudi

      Non-Saudi

4.     Medical Degree

      Resident

      Registrar

      Consultant

5.     Zone

      Zone One

      Zone Two

      Zone Three

      Zone Four

      Zone Five

6.     Years in practice

a.     ≤ 5 

b.     6-10

c.     11-15

d.     >15

Part II: Knowledge 

7.     For the majority of your asymptomatic average-risk patients, you will start screening at age of:

      45 years

      50 years

      55 years

      60 years

      Not Sure 

8.     Is there an age at which you no longer recommend screening for healthy patients?

      No

      Yes, at: _______ years of age.

9.     The frequency of screening with FOBT is every:

      One year

      Two years

      Three years

      Not sure

10.  Are you aware of the following types of FOBT?

a.     Guaiac FOBT

o   Yes

o   No 

b.     Fecal Immunochemical Testing

o   Yes

o   No 

11.  Are you aware of the following means of conducting FOBT?

a.     FOBT card in the office during digital rectal exam

o   Yes

o   No

b.     Give patients FOBT kits to complete at home

o   Yes

o   No 

12. For Colorectal Cancer Screening using FOBT, how many samples do you suppose to order?

      One

      Two

      Three

      Not sure

13.  The frequency of screening with Sigmoidoscopy is every:

      1 year

      3 years

      5 years

      10 years

      Not sure

14.  The frequency of screening with Colonoscopy is every:

      1 year

      3 years

      5 years

      10 years

      Not sure

Part III: Attitude

15.  Do you think that Colorectal Cancer Screening for asymptomatic average-risk patients aged 45 years and older is effective?

      Yes

      No 

16.  How effective do you believe the following screening procedures are in reducing colorectal cancer mortality in average-risk patients aged 45 years and older? 

a.     Fecal Occult Blood Testing

o   Effective

o   Not Effective

o   Don’t Know

b.     Flexible Sigmoidoscopy

o   Effective

o   Not Effective

o   Don’t Know

c.     Colonoscopy

o   Effective

o   Not Effective

o   Don’t Know

d.     Double-Contrast Barium enema

o   Effective

o   Not Effective

o   Don’t Know

e.     CT-Colonography

o   Effective

o   Not Effective

o   Don’t Know

Part IV: Practice

17.  Do you recommend and advise asymptomatic average-risk patients aged 45 years and older for Colorectal Cancer Screening? 

      Yes

      No 

18.  Which of the following screening tests do you recommend for your asymptomatic average-risk patients?(CHECK ALL THAT APPLY)

      Fecal Occult Blood Test

      Sigmoidoscopy

      Colonoscopy

      Other (specify): _______________        

19.  During a typical month, how many times do you refer asymptomatic, average-risk patients for colorectal cancer screening?

      0 times

      1-5 times

      6-10 times

      11-20 times

      >20

20.  When you talk to your asymptomatic, average-risk patients about colorectal cancer screening, how often do you encounter the following?

a.     Not having enough time to discuss screening with my patients

o   Never

o   Rarely

o   Sometimes

o   Usually

b.     My patients do not want to discuss colorectal cancer screening

o   Never

o   Rarely

o   Sometimes

o   Usually

c.     My patients have difficulty understanding the information I present about colorectal cancer screening

o   Never

o   Rarely

o   Sometimes

o   Usually

d.     My patients are unaware of colorectal cancer screening

o   Never

o   Rarely

o   Sometimes

o   Usually

e.     My patients do not perceive colorectal cancer as a serious health threat

o   Never

o   Rarely

o   Sometimes

o   Usually

Other (specify): __________________
